# LncRNA CTBP1-AS2 regulates miR-216a/ PTEN to suppress ovarian cancer cell proliferation

**DOI:** 10.1186/s13048-020-00689-6

**Published:** 2020-07-25

**Authors:** Kaiying Cui, Genhai Zhu

**Affiliations:** Department of Gynaecology, Hainan People’s Hospital, Hainan Province, Haikou City, 570311 PR China

**Keywords:** CTBP1-AS2, Ovarian cancer, miR-216a, PTEN, Proliferation

## Abstract

**Background:**

We analyzed TCGA dataset and observed the downregulation of CTBP1-AS2 in ovarian cancer (OC), while the function of CTBP1-AS2 has only been investigated in diabetes and cardiomyocyte hypertrophy, but not in cancer biology. We therefore analyzed the involvement of CTBP1-AS2 in OC.

**Result:**

We found that CTBP1-AS2 was downregulated in OC and predicted poor survival. CTBP1-AS2 in luciferase activity assay interacted with miR-216a, while overexpression of CTBP1-AS2 and miR-216a had no significant effects on the expression of each other. However, increased expression level of PTEN, a target of miR-216a, was observed after CTBP1-AS2 overexpression. Increased proliferation rate of OC cells was observed after the overexpression of miR-216a. CTBP1-AS2 and PTEN overexpression resulted in the reduced proliferation rate of OC cells and reduced effects of miR-216a overexpression.

**Conclusion:**

CTBP1-AS2 regulates miR-216a/PTEN to suppress OC cell proliferation.

## Introduction

Ovarian cancer (OC) is a commonly diagnosed female malignancy in clinical practice [1]. The latest GLOBOCAN reported that OC in 2018 caused a total number of 184,799 deaths, accounting for 1.9% of all cancer-related deaths [2]. In the same year, a total of 295,414 new cases of OC were diagnosed, which were the 1.6% of all new cancer cases [2]. OC patients at early stages show no symptoms or mild symptoms. Therefore, most OC patients are diagnosed at advanced stages [3, 4]. Although overweight, diabetes and smoking have been reported to be closely correlated with the occurrence of OC, pathogenesis of this disease remains unclear [5, 6]. Therefore, in-depth analysis of the molecular mechanism is needed to improve the development of novel anti-OC therapy.

Studies on the molecular pathogenesis of OC have identified a considerable number of molecular pathways involved in the pathogenesis of this disease [7, 8]. The functional analysis of these molecular players in OC accelerates the development of targeted therapy, which aims to suppress cancer progression by regulating cancer-related gene expression [9, 10]. Long non-coding RNAs (lncRNAs) have no capacity of protein-coding but they regulate cancer development by regulating gene expression at multiple levels [10]. In effect, regulating the expression of lncRNAs now is considered as a potential target for cancer treatment [11, 12]. However, the role of most lncRNAs in cancer biology remains unclear. LncRNA CTBP1-AS2 is a recently characterized crucial player in diabetes and cardiomyocyte hypertrophy [13, 14], while its role in cancer biology remains unclear. We analyzed TCGA dataset and observed the downregulation of CTBP1-AS2 in OC. In addition, CTBP1-AS2 is predicted to interact with miR-216a, which can target PTEN to play oncogenic roles [15]. This study aimed to analyze the interactions between CTBP1-AS2, miR-216a and PTEN in OC.

## Materials and methods

### OC patients and tissue collections

This study was approved by the Ehics Committee of Hainan People’s hospital. Study patients of this study were 60 OC patients (age: 37 to 67 years; mean ± S.D. age: 54.1 ± 6.6 years) who were enrolled at aforementioned hospital betweem January 2012 and December 2014. All patients were excluded from other clinical disorders and no therapy was performed on these patients before this study. Patients with a previous history or familly history of malignancies were also excluded. Ovarian biopsy was perfromed on all 60 patients before therapy to collect both adjacent (within 5 cm around tumor) non-tumor avarian tissues and OC tissues. Histopathological analysis was performed to confirm correct tissue samples were obtained. All patients signed informed consent.

### Treatment and follow-up

According to AJCC system, the 60 patients included 10, 13, 21 and 16 cases at clinical stage I, II, III and IV, respectively. Therapeutic approaches, such as surgical resections, chemotherapy, radiotherapy, and immunotherpay, were performed on these patients according to patients’ clinical stage and health conditions. All patients were followed up for 5 years from the day of admission. Patients’ survival conditions were recorded. All patients completed the 5 year follow-up.

### Cell culture and transfection

Human OC cell line UWB1.289 from ATCC (USA) was used. Cell culture medium was composed of 10% FBS and 90% 1:1 mixture of RPMI-1640 medium/ MEGM medium. Cells were cultivated in a 5% CO_2_ incubator at 37 °C with 95% humidity. Subsequent experiments were performed 48 h later.

### Cell transfections

The construction of expression vectors of CTBP1-AS2 and PTEN was performed using pcDNA3.1 vector (Sigma-Aldrich) as backbone. Mimic of miR-216a and negative control (NC) miRNA were from Invitrogen. UWB1.289 cells were transfected with 10 nM expression vector and/or 50 nM miRNA using Lipofectamine 2000 (Invitrogen, USA). All steps were completed according to manufacturer’s instructions. For controls, cells transfected with empty vector or NC miRNA were NC cells, and untransfected cells were control (C) cells. Subsequent experiments were performed 48 h later.

### Dual luciferase activity assay

pGL3 vector (Promega Corporation) was used as backbone to establish the luciferase vector of CTBP1-AS2. To analyze the interaction between CTBP1-AS2 and miR-216a, UWB1.289 cells were co-transfected with NC miRNA+ CTBP1-AS2 luciferase vector (NC group) or miR-216 mimic + CTBP1-AS2 luciferase vector (miR-216a group) through aforementioned methods. At 48 h post-transfection, luciferase activities of both groups were measured and compared.

### RNA isolations

Isolation of total RNAs from OC tissues, non-tumor tissues, and UWB1.289 cells was performed using Ribozol (Sigma-Aldrich). RNA precipitation was performed using 85% ethanol to harvest miRNAs. Genomic DNA was removed by DNase I digestion at 37 °C for 2 h.

### RT-qPCR

The synthesis of cDNA samples was performed using SSRT IV kit (Thermo Fisher Scientific) with total RNA as template. SYBR Green Master Mix (Bio-Rad) was used to perform all qPCR reactions with GAPDH internal control to measure the levels of CTBP1-AS2 and PTEN mRNA expression. To measure the levels of miR-216a expression, poly (A) addition, reverse transcriptions and qPCR reactions were performed using All-in-OneTM miRNA qRT-PCR Detection Kit (GeneCopoeia) with U6 as the internal control of miR-216a. Three replicate reactions were involved in each experiment and gene expression levels were normalized using 2^-ΔΔCq^ method.

### Western-blot assay

The isolation of total protein from UWB1.289 cells was performed using RIPA buffer (Invitrogen). Protein concentrations were measured by performing BCA assay (Invitrogen). Protein samples were denatured in boiling water for 10 min, followed by separation of proteins using SDS-PAGE gel (8%). Proteins were transferred to PVDF membranes, followed by blocking in PBS containing 5% non-fat milk for 2 h at 25 °C. The blocked membranes were first incubated with rabbit primary antibodies of PTEN (ab31392,Abcam) and GAPDH (ab9485, Abcam) at 4 °C for 20 h, followed by incubation with secondary antibody of lgG-HRP (ab6721, Abcam) for 2 h at 25 °C. After that, signals were developped using ECL™ Select Western Blotting Detection Reagent (Sigma-Aldrich). Image J v1.48 software was used to normalize signals.

### CCK-8 assay

A Cell Counting Kit-8 (CCK-8) kit from Dojindo (Japan) was used to analyze the proliferation of UWB1.289 cells after transfections. UWB1.289 cells were cultivated in a 96-well plate with 4000 cells in 0.1 ml medium per well. Cells were cultivated at 37 °C and CCK-8 solution was added to reach 10% final concentration before the measurement of OD values. OD values were measured at 450 nM every 24 h for a total of 96 h.

### Statistical analysis

All experiments were carried out in 3 independent biological replicates. Data were expressed as mean ± S.D. values. Paired OC and non-tumor tissues were compared by paired t test. Unpaired t test was used to compare two independent groups. ANOVA Tukey’s test was used to compare multiple groups. With the median expression level of CTBP1-AS2 in OC tissues as cutoff value, the 60 patients were divided into high and low CTBP1-AS2 level groups (*n* = 30). Survival curves were plotted for both groups and compared by log-rank test. *p* < 0.05 was statistically significant.

## Results

### Downregulation of CTBP1-AS2 in OC predicted poor survival

**Fig. 1:**
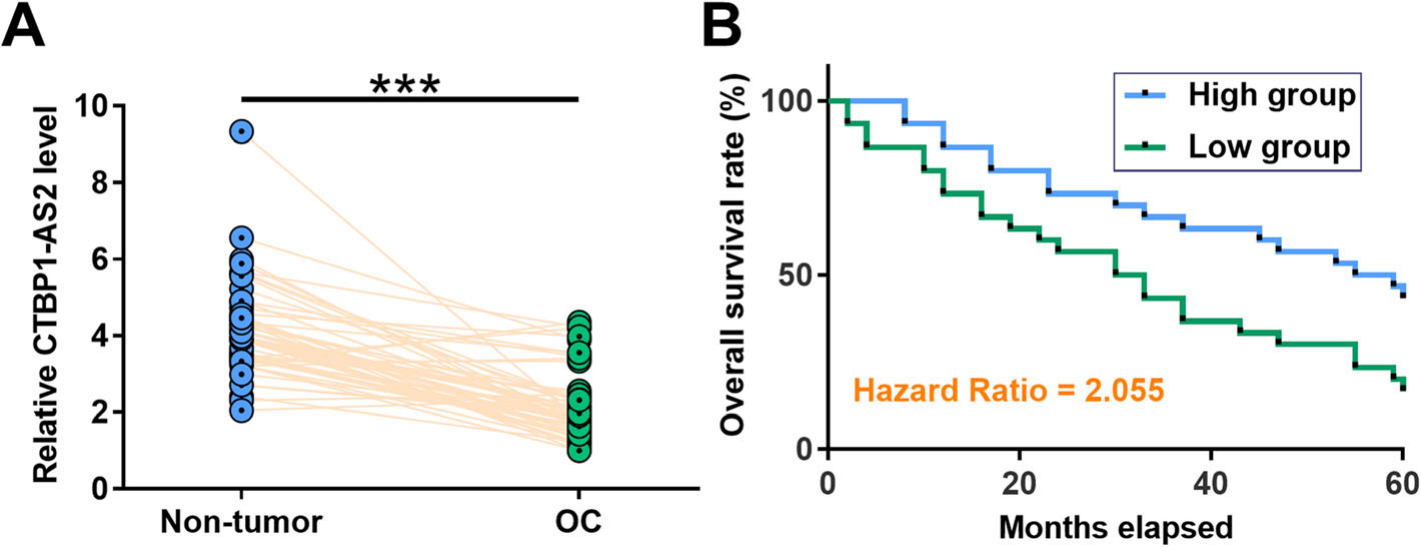
Downregulation of CTBP1-AS2 in OC predicted poor survival. Expression levels of CTBP1-AS2 in paired OC and non-tumor tissues from 60 OC patients included in this study were measured by RT-qPCR. PCR reactions were repeated 3 times and mean values were compared (**a**). With the median expression level of CTBP1-AS2 in OC tissues as cutoff value, the 60 patients were divided into high and low CTBP1-AS2 level groups (*n* = 30). Survival curves were plotted for both groups and compared by log-rank test (**b**)

TCGA dataset was explored to analyze the differential expression of CTBP1-AS2 in OC. It was observed that expression level of CTBP1-AS2 was higher in OC tissues in comparison to non-tumor tissues (6.55 vs.8.47). To confirm its downregulation in OC, expression levels of CTBP1-AS2 in paired OC and non-tumor tissues from 60 OC patients included in this study were measured by RT-qPCR. Compared with non-tumor tissues, significantly lower levels of CTBP1-AS2 expression were observed in OC tissues (Fig.1A, *p* < 0.001). Survival curves were plotted for both high and low CTBP1-AS2 level groups (*n* = 30). Compared with patients in high level group, patients in low CTBP1-AS2 level group showed significantly higher mortality rate (Fig.1B).

### CTBP1-AS2 interacted with miR-216a in UWB1.289 cells

**Fig. 2:**
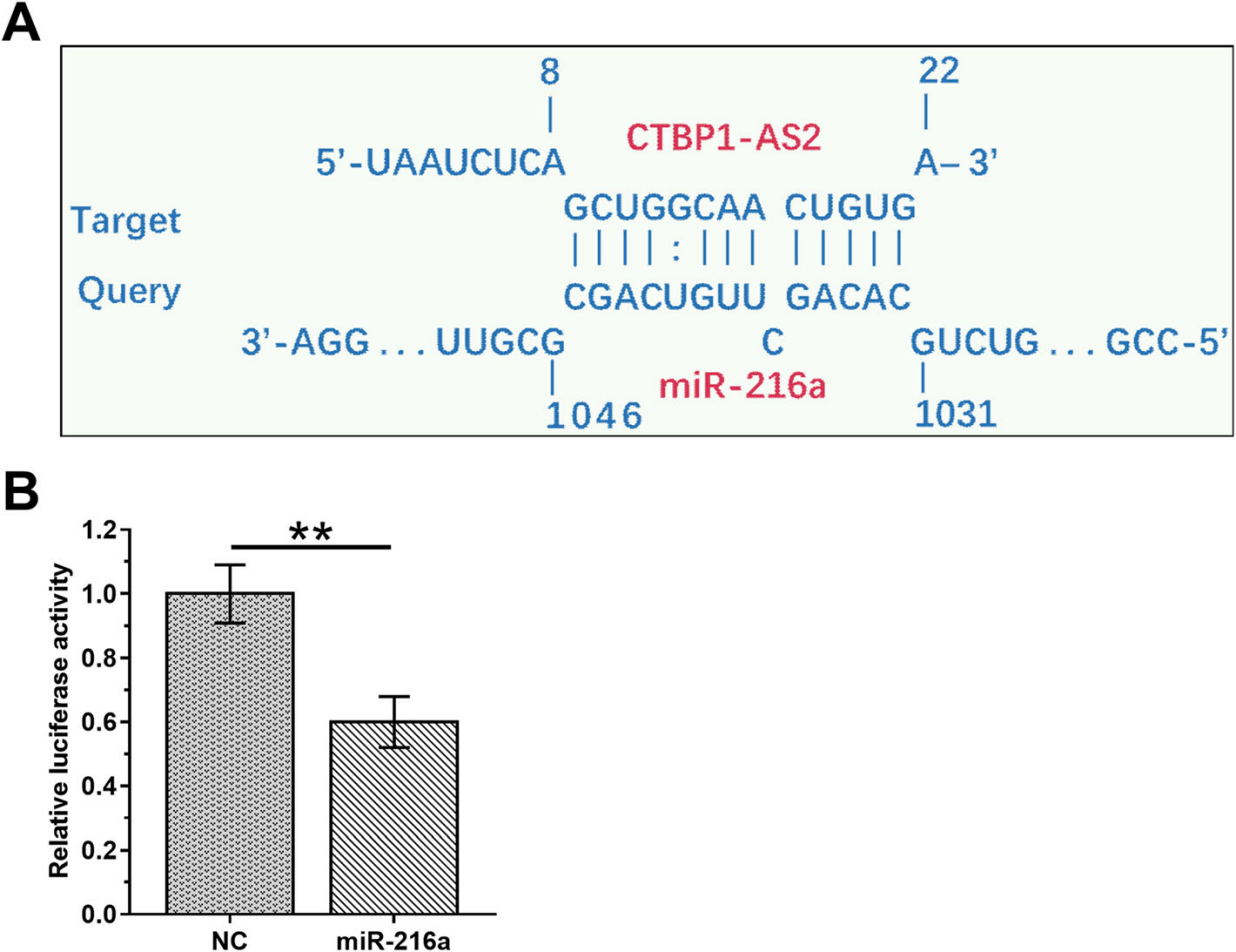
CTBP1-AS2 interacted with miR-216a in UWB1.289 cells. The interaction between CTBP1-AS2 and miR-216a was predicted by IntaRNA2.0 (**a**). To further confirm the interaction, UWB1.289 cells were co-transfected with NC miRNA+ CTBP1-AS2 luciferase vector (NC group) or miR-216 mimic + CTBP1-AS2 luciferase vector (miR-216a group). This experiment was repeated 3 times and mean ± S.D. values were compared.*,*p* < 0.01

The interaction between CTBP1-AS2 and miR-216a was predicted by IntaRNA2.0 (http://rna.informatik.uni-freiburg.de/IntaRNA/). It was observed that CTBP1-AS2 and miR-216a may form multiple base pairs (Fig.2A). To further confirm the interaction, UWB1.289 cells were co-transfected with NC miRNA+ CTBP1-AS2 luciferase vector (NC group) or miR-216 mimic + CTBP1-AS2 luciferase vector (miR-216a group). Compared with NC group, significantly lower luciferase activity was observed in miR-216a group (Fig.2B, *p* < 0.05).

### CTBP1-AS2 and miR-216a failed to regulate the expression of each other

**Fig. 3:**
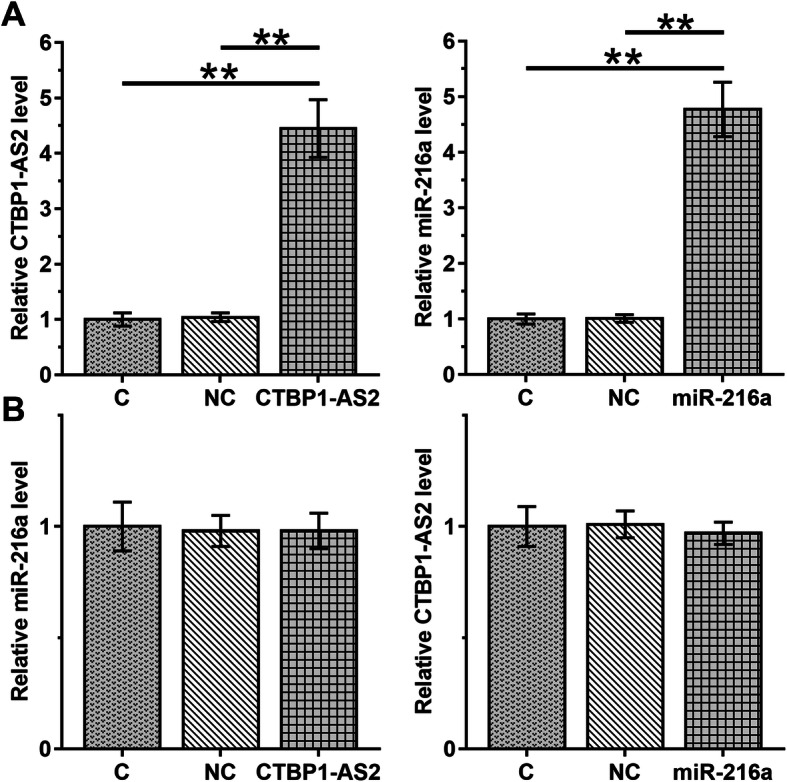
CTBP1-AS2 and miR-216a failed to regulate the expression of each other. To further analyze the interaction between CTBP1-AS2 and miR-216a, UWB1.289 cells were transfected with CTBP1-AS2 expression vector or miR-216a mimic, and the overexpression of CTBP1-AS2 and miR-216a was confirmed by RT-qPCR (**a**). The effects of CTBP1-AS2 and miR-216a overexpression on the expression of each other were also analyzed by RT-qPCR (**b**). Experiments were repeated 3 times and mean ± S.D. values were compared.*,*p* < 0.01

To further analyze the interaction between CTBP1-AS2 and miR-216a, UWB1.289 cells were transfected with CTBP1-AS2 expression vector or miR-216a mimic, and the overexpression of CTBP1-AS2 and miR-216a was confirmed by RT-qPCR (Fig.3A, *p* < 0.01). Compared with C and NC groups, overexpression of CTBP1-AS2 and miR-216a failed to affect the expression of each other (Fig.3B).

### CTBP1-AS2 overexpression led to upregulated PTEN in UWB1.289 cells

**Fig. 4:**
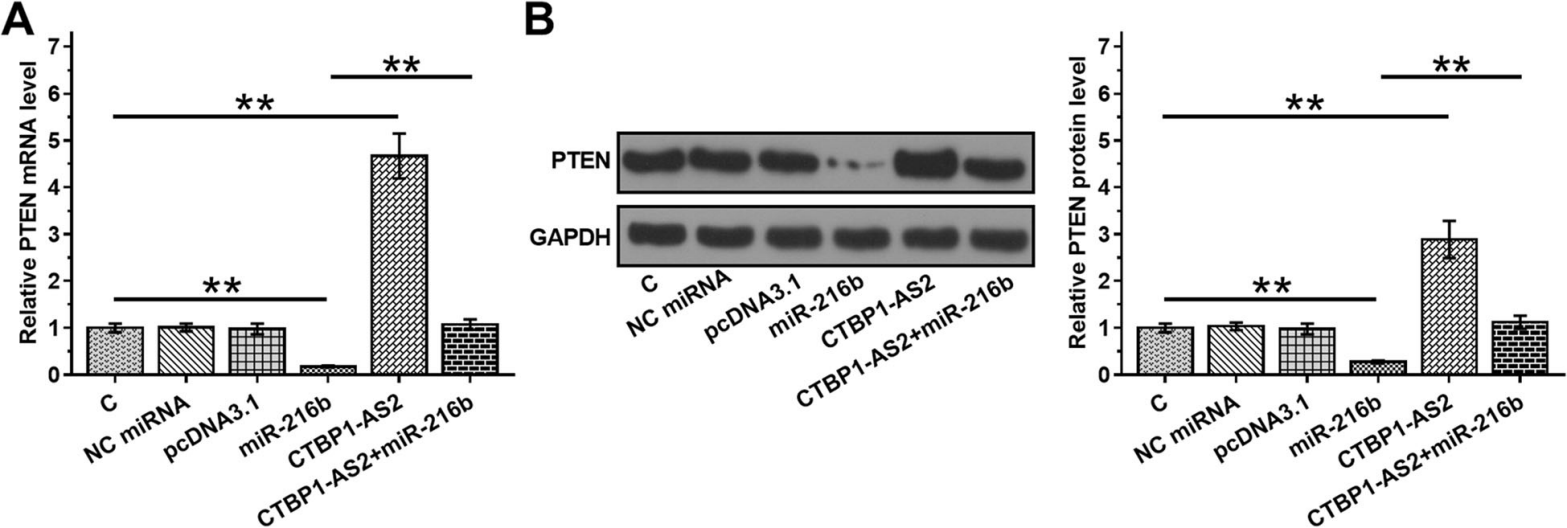
CTBP1-AS2 overexpression led to upregulated PTEN in UWB1.289 cells. To test the possibility that CTBP1-AS2 may sponge miR-216a, the effects of CTBP1-AS2 and miR-216a overexpression on the expression of PTEN expression in UWB1.289 cells were analyzed by RT-qPCR (**a**) and western blot (**b**). Experiments were repeated 3 times and mean ± S.D. values were compared.*,*p* < 0.01

To test the possibility that CTBP1-AS2 may sponge miR-216a, the effects of CTBP1-AS2 and miR-216a overexpression on the expression of PTEN expression in UWB1.289 cells were analyzed by RT-qPCR (Fig.4A) and western blot (Fig.4B). It was observed that miR-216a overexpression resulted in the downregulation of PTEN (*p* < 0.01). In contrast, CTBP1-AS2 overexpression played an opposite role and reduced the effect of miR-216a overexpression (*p* < 0.01).

### CTBP1-AS2 regulated miR-216a/PTEN axis to suppress cell proliferation

**Fig. 5:**
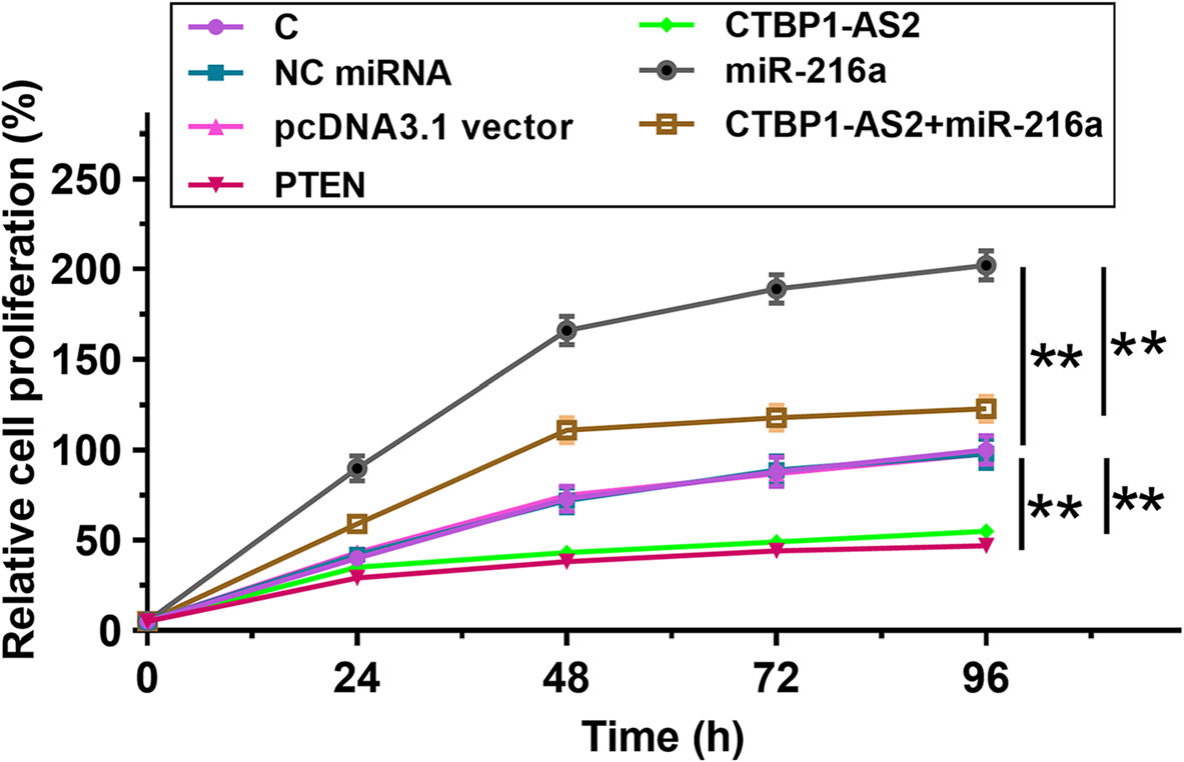
CTBP1-AS2 regulated miR-216a/PTEN axis to suppress cell proliferation. The role of CTBP1-AS2, miR-216a and PTEN in regulating OC cell proliferation was analyzed by performing CCK-8 assay. Experiments were repeated 3 times and mean ± S.D. values were compared.*,*p* < 0.01

The role of CTBP1-AS2, miR-216a and PTEN in regulating OC cell proliferation was analyzed by performing CCK-8 assay. Compared with C group, increased proliferation rate of OC cells was observed after the overexpression of miR-216a (Fig.5, *p* < 0.01). CTBP1-AS2 and PTEN overexpression resulted in the reduced proliferation rate of OC cells (*p* < 0.01). In addition, CTBP1-AS2 overexpression reduced effects of miR-216a overexpression (*p* < 0.01).

## Discussion

In this stud the interactions between CTBP1-AS2, miR-216a and PTEN were analyzed in OC. It was observed that CTBP1-AS2 was downregulated in OC. In addition, CTBP1-AS2 may serve as an internal spongy of miR-216a to upregulate PTEN, thereby suppressing OC cell proliferation.

Based on our knowledge, the role of CTBP1-AS2 has only be investigated in type 2 diabetes and cardiomyocyte hypertrophy [13, 14]. It was observed that the low level of CTBP1-AS2 expression in peripheral blood mononuclear cells is closely correlated with the high risk factor of type 2 diabetes [13]. In cardiomyocyte hypertrophy, CTBP1-AS2 stabilizes TLR4 by interacting with FUS, thereby regulating the development of disease [14]. By analyzing TCGA dataset and measuring the levels of CTBP1-AS2 in paired OC and non-tumor tissues from 60 OC patients our study first reported the downregulation of CTBP1-AS2 in OC. In addition, CTBP1-AS2 overexpression led to decreased proliferation of OC cells. Therefore, CTBP1-AS2 may play tumor suppressive role in OC. In effect, by analyzing TCGA dataset we observed the altered expression of CTBP1-AS2 in multiple types of cancers. The involvement of CTBP1-AS2 in other types of cancers remains to be further investigated.

MiR-216a plays different roles in different types of cancers [16, 17]. For instance, miR-216a targets CDK14 to suppress the proliferation and invasion of osteosarcoma cells [16]. In contrast, miR-216a targets PTEN/AKT pathway to suppress the metastasis of OC [17]. Consistently, our study confirmed the oncogenic role of miR-216a in OC. It has been reported that miR-216a can target PTEN to promote liver cancer [15]. Our study showed that miR-126a may also target PTEN in OC to promote the proliferation of OC cells. Therefore, miR-216a may target multiple oncogenes in OC.

Our study showed that miR-216a and CTBP1-AS2 could interact with each other, while overexpression experiments showed that they could not regulate the expression of each other. Instead, CTBP1-AS2 overexpression reduced the inhibitory role of CTBP1-AS2 in regulating PTEN expression as well as its enhancing role in regulating OC cell proliferation. Therefore, CTBP1-AS2 may sponge miR-216a.

In conclusion, CTBP1-AS2 is downregulated in OC. I CTBP1-AS2 may sponge miR-216a to upregulate PTEN, thereby suppressing OC cell proliferation.

## Data Availability

The datasets used and analyzed during the current study are available from the corresponding author on reasonable request.
